# Using WhatsApp and Facebook Online Social Groups for Smoking Relapse Prevention for Recent Quitters: A Pilot Pragmatic Cluster Randomized Controlled Trial

**DOI:** 10.2196/jmir.4829

**Published:** 2015-10-22

**Authors:** Yee Tak Derek Cheung, Ching Han Helen Chan, Chi-Keung Jonah Lai, Wai Fung Vivian Chan, Man Ping Wang, Ho Cheung William Li, Sophia Siu Chee Chan, Tai-Hing Lam

**Affiliations:** ^1^ School of Public Health The University of Hong Kong Hong Kong China (Hong Kong); ^2^ Integrated Centre on Smoking Cessation Tung Wah Group of Hospitals Hong Kong China (Hong Kong); ^3^ School of Nursing The University of Hong Kong Hong Kong China (Hong Kong)

**Keywords:** social networking, social media, smoking cessation, relapse prevention

## Abstract

**Background:**

Quit attempters often have episodes of smoking relapse before they eventually quit. Interactive text messaging through mobile phones has been shown to increase abstinence. This service can be potentially applied on the platform of a social networking service to help quitters maintain abstinence.

**Objective:**

Our aim was to determine if the group discussion and reminders via the WhatsApp or Facebook social group were effective to prevent smoking relapse in quitters who had stopped smoking recently.

**Methods:**

This was a single-blinded, parallel, 3-arm pilot cluster randomized controlled trial allocating recent quitters, who had completed an 8-week treatment and reported abstinence for at least 7 days, to WhatsApp (n=42), Facebook (n=40), and a control group (n=54). The 2 intervention groups participated in a 2-month online group discussion with either WhatsApp or Facebook moderated by a trained smoking cessation counselor and received a self-help booklet on smoking cessation. The control group only received the booklet. The primary outcome was the 2- and 6-month relapse rates, defined as the proportion of participants who smoked at least 5 cigarettes in 3 consecutive days.

**Results:**

Fewer participants in the WhatsApp group (17%, 7/42) reported relapse than the control group (42.6%, 23/54) at 2-month (OR 0.27, 95% CI 0.10-0.71) and 6-month (40.5%, 17/42 vs 61.1%, 33/54; OR 0.43, 95% CI 0.19-0.99) follow-ups. The Facebook group (30.0%, 12/40) had an insignificantly lower relapse rate than the control group (42.6%, 23/54) at 2-month (OR 0.58, 95% CI 0.24-1.37) and 6-month (52.5%, 13/40 vs 61.1%, 33/54; OR 0.70, 95% CI 0.31-1.61) follow-ups. The WhatsApp social groups had more moderators’ posts (median 60, IQR 25 vs median 32, IQR 7; *P*=.05) and participants’ posts (median 35, IQR 50 vs median 6, IQR 9; *P*=.07) than their Facebook counterparts, but the difference was insignificant.

**Conclusions:**

The intervention via the WhatsApp social group was effective in reducing relapse probably because of enhanced discussion and social support. Inactive discussion in the Facebook social group might have attributed to the lower effectiveness.

**ClinicalTrial:**

Clinicaltrials.gov NCT02007369; https://clinicaltrials.gov/show/NCT02007369 (Archived by WebCite® at http://www.webcitation.org/6c3RbltQG)

## Introduction

The World Health Organization’s MPOWER measures includes “offer help to quit tobacco use” as one of 6 effective tobacco control strategies [1]. Despite the availability of medication and counseling services, quit attempters often have smoking “slips” (ie, one or a few puffs) or relapses before sustaining longer abstinence [2]. Quitters who quit smoking recently have to manage nicotine withdrawal symptoms and smoking cues in their daily environment. Approximately one-third of quitters relapse smoking 3 months after completing smoking cessation treatment and this proportion is 50% for those who quit for a week or less [3]. A US longitudinal study of smokers who received smoking cessation pharmacotherapies in primary care clinics found that approximately 80% relapsed smoking within a year after the treatment [4].

A meta-analysis of relapse prevention interventions showed that smoking cessation drugs to reduce nicotine cravings and withdrawal symptoms, such as bupropion (pooled OR 1.49, 95% CI 1.10-2.01) and nicotine replacement therapy (NRT) (pooled OR 1.33, 95% CI 1.08-1.63) were effective in preventing smoking relapse for at least 12 months [5]. However, the prevalence of use was low [6] and the compliance was poor (ie, use for less than 8 weeks) [7]. Previous randomized controlled trials (RCTs) using individual counseling or self-help written materials had inadequate sample size to support the effectiveness of relapse prevention [8]. Group counseling was effective for preventing relapse in the short term (eg, 3 months) (pooled OR 2.55, 95% CI 1.58-4.11), but the effect dissipated at long-term follow-up [5]. The limited effect might be explained by only a few face-to-face group sessions [9-11], which failed to offer instant and continuing support for recent quitters to manage craving or smoking cues.

Mobile phone-based interventions are potentially effective to support recent quitters to quit [12,13] and prevent relapse [14,15]. In Hong Kong, with approximately 7 million residents, there are more than 17 million subscribers to mobile phone services and more than 12 million of them are 2.5G/3G/4G subscribers with mobile Internet services [16]. Because Internet access with mobile phones has become popular, interventions via social networking services to support health-related behavior change have been examined for weight control and increasing physical activity [17]. A systematic review showed that such interventions have small to moderate effect size (-0.05 to 0.84), with only 2 of 7 studies showing statistically significant effects [17]. A few exploratory studies showed that a social networking service enabled reaching a sizable number of smokers in the community and increased peer interaction [18-21]. It can be a platform for smokers who seek immediate assistance and professional advice when they need it [22].

In this pilot RCT, we tested the effectiveness of a relapse prevention intervention using WhatsApp and Facebook, 2 common mobile phone apps in Hong Kong, to reduce smoking relapse in recent quitters who had just completed treatment at smoking cessation clinics.

## Methods

### Trial Design

The pilot single-blinded, pragmatic, parallel 3-armed cluster RCT compared the relapse rate at 2- and 6-month follow-ups between recent quitters who participated in group discussion and received reminders (group A: WhatsApp; group B: Facebook) and those who did not (group C: control; allocation ratio 1:1:1). The study was approved by the Institutional Review Board of the University of Hong Kong / Hong Kong Authority Hong Kong West Cluster (IRB reference no: UW-13-528).

### Participants

All participants were clients of the Tung Wah Group of Hospitals Integrated Centre of Smoking Cessation (ICSC) in China Hong Kong, which provides 8-week free treatment, including counseling, telephone follow-ups, physicians’ assessment, and prescription of free NRT or varenicline (a smoking cessation drug to relieve cravings while blocking the reinforcing effects of nicotine) [23]. At 8-week follow-up during telephone or face-to-face counseling, clients were asked by the ICSC counselors if they had quit. Self-reported quitters were then screened with the criteria for eligibility, including (1) daily smoker at first entry to the ICSC, (2) aged 18 years or older, (3) received 3 to 8 smoking cessation counseling sessions provided by the ICSC, (4) reported tobacco abstinence for at least 7 days, (5) able to communicate in Cantonese and read and write Chinese, (6) had a mobile phone through a local network, and (7) were able to access the Internet by mobile phone. Clients were excluded who had unstable psychological conditions as advised by physicians, possible alcohol dependence as measured by the Alcohol Use Disorders Identification Test (AUDIT) [24], or were pregnant.

### Interventions

Treatment conditions for groups A and B included participation in the WhatsApp or Facebook online social group, respectively, and a 22-page booklet related to quitting and healthy diet. The social group function of WhatsApp and Facebook was used as the intervention platform because it supports a real-time sharing of text and multimedia messages among group members. Each social group started on the first day after each recruitment week and closed after 2 months. Group members received 3 reminders per week from a moderator who was a social worker or nurse with experience in smoking cessation counseling. These reminders, including texts, pictures, and videos, were based on the “Treatments for the Recent Quitter” of the US Clinical Practice Guidelines on Treating Tobacco Use and Dependence [2], including (1) encourage to maintain abstinence, (2) remind about the importance of remaining abstinence, (3) prevent smoking triggers, (4) remind about the withdrawal symptoms and lapse, (5) advise about stress and mood management, and (6) advise about weight control (Multimedia Appendix 1). All moderators were provided a guideline in sending reminders, enhancing discussion, and other tasks in the social group (Multimedia Appendix 2).

Participants’ privacy was protected by advising them to change the privacy setting in their WhatsApp and Facebook accounts and setting up participation rules. Because telephone numbers appear in the WhatsApp social groups, male and female participants in the WhatsApp group were separated into different social groups to avoid the possibility of misconduct or harassment, which was a concern raised by some female respondents in our pilot qualitative interviews. Telephone numbers can be concealed in Facebook; therefore, sex separation was not applied. Group C was a control group; they received only the same self-help booklet and were advised to contact ICSC’s counselors when they faced high-risk situations or had smoking lapses (usual care).

### Follow-Up

All participants were contacted via telephone at 2- and 6-month follow-ups after the random group allocation. Interviewers were blinded to the group assignment. Participants who reported tobacco abstinence in the past 7 days were visited by trained staff to collect their exhaled carbon monoxide (CO) and saliva sample. The participant was given HK $100 (approximately US $12.80) if his/her exhaled CO was less than 4 parts per million (ppm) and salivary cotinine was less than 10 ng/mL, which confirmed abstinence [25,26]. To minimize the incentive effect, if any, on the validation result, the incentive was small, only enough to compensate for travel and a little time cost. All participants were unaware of the incentive before follow-up and only the participants who reported abstinence were notified of the incentive.

### Outcomes

The primary outcome was self-reported relapse rate, which was defined as the proportion of participants who self-reported smoking at least 5 cigarettes in 3 consecutive days in the past 2 months at the 2-month follow-up [27]. Another primary outcome was the 4-month relapse rate at 6-month follow-up. Secondary outcomes included (1) self-reported any smoking incidence (ie, smoking lapse), (2) self-reported smoking in the past 7 days, and (3) biochemically validated abstinence at the 2 follow-ups.

The questionnaire also collected other smoking-related information, including frequency of smoking urges in the past week [28], intensity of smoking urge in the past 24 hours [29], thinking of enjoying smoking [28], the Minnesota Nicotine Withdrawal Scale (MNWS) [29], and the 12-item Smoking Self-Efficacy Questionnaire (SEQ-12) [30].

### Sample Size

The expected sample size was 40 for each group (ie, total sample size=120) to generate preliminary estimates of the intervention effectiveness. ICSC’s treatment record showed for quitters at the RCT enrollment, approximately one-third reported a smoking relapse at 6-month follow-up. Assuming the quit rate of group C was 33.3% and the effect size of the primary outcome between groups A/B and C was 1.5, the estimated relapse rates for groups A/B and C were approximately 22% and 33%, respectively. The power for detecting this difference in 120 participants using the Fisher exact test is 22%, suggesting we might wrongly accept the null hypothesis (ie, no difference between groups A/B and C).

### Randomization

Cluster randomization was used to allocate all participants recruited in a particular week to one RCT group. This randomization could allocate a sufficient number of participants in a social group each week and the selection bias due to recruitment week was unlikely. The estimated recruitment period was 9 weeks and each week was randomized to group A, B, and C using numbers generated on a website for generating random variables (RANDOM.ORG) by one of the authors (CYTD). After the 9-week recruitment, the number of participants in groups B and C were only 19 and 27, respectively. Therefore, we extended the recruitment period by 5 additional weeks and used the same randomization method.

### Concealment Mechanism

The ICSC counselors who screened and enrolled participants were notified of the group allocation on Monday of each recruitment week. Participants were not aware of the allocation sequence.

### Blinding

All participants received a specific relapse prevention intervention, but they did not know what the other interventions were. All assessors of outcomes were blinded to the RCT group of each participant.

### Statistical Analysis

Data were entered into SPSS for Windows version 20 for analysis. Descriptive statistics including frequency, percentage, and mean were used to summarize the outcomes and other variables. Chi-square tests were used to compare categorical variables between subgroups. The Kolmogorov-Smirnov test was used to determine the use of *t* test (normal distribution) or Mann-Whitney *U* test (nonnormal distribution) for the comparison. We analyzed the primary and secondary outcomes with Fisher exact test and odds ratios with and without adjustment for significantly different characteristics at baseline. By intention-to-treat (ITT) analysis [31], participants who were lost or refused to follow up were treated as having smoking relapse and lapse. Sensitivity analysis assuming that missing participants had not changed smoking status since the previous follow-up (last observation carried forward [LOCF]) and excluding participants who were lost to follow-up (complete-case analysis) were performed for the primary outcomes. Additional analysis excluding those in groups A and B who did not participate in the social groups was also conducted. General linear model repeated measures analysis was used to examine the changes of other smoking-related variables.

A content analysis of all the posts in the social groups was conducted to understand how the intervention helped participants prevent relapse. All posts in the WhatsApp and Facebook social groups were archived before the social groups were closed by the moderator. Each post was coded by 2 researchers separately and was classified by their content. The Mann-Whitney *U* test was used to compare the median number of posts between the WhatsApp and Facebook social groups because we had no assumption about their statistical distribution.

## Results

### Group Allocation and Retention Rates at Follow-Ups

**Figure 1:**
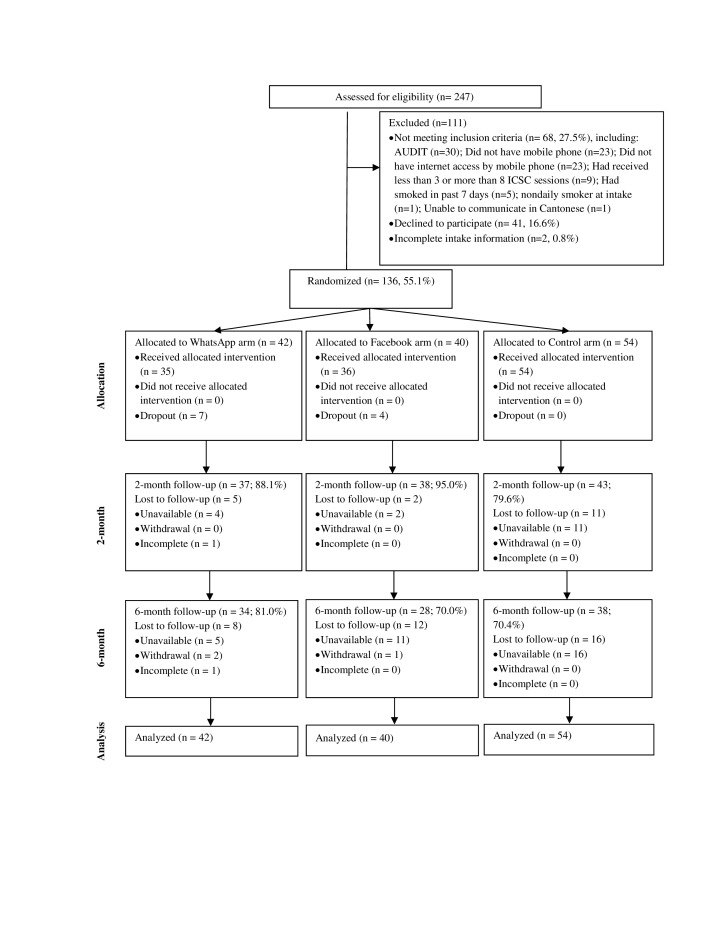
CONSORT flow diagram of the pilot randomized controlled trial. AUDIT: The Alcohol Use Disorders Identification Test.

From February 2014 to May 2014, 247 quitters were screened for eligibility. Of these, 68 quitters (27.5%) were ineligible, 41 (16.6%) refused to participate, and 2 (0.8%) had incomplete intake information. In all, 136 quitters (55.1%) participated with 42 allocated to group A (WhatsApp), 40 to group B (Facebook), and 54 to group C (Control) (Figure 1). The major reasons for ineligibility were possible alcohol dependence measured by AUDIT (n=30), had no mobile phone (n=23), or no Internet access by mobile phone (n=23).

For the 136 participants, 86.8% (118/136) were successfully followed at 2-month follow-up with 88% (35/42) in group A, 95% (36/40) in group B, and 80% (43/54) in group C (Figure 1). The overall retention rate at 6-month follow-up was 73.5% (100/136), with 81% (34/42) in group A, 70% (28/40) in group B, and 70% (38/54) in group C. The reasons for loss to follow-ups were (1) unable to reach via telephone, (2) refusal to follow up, and (3) incomplete survey.

### Demographic Characteristics and Smoking Profile

For the 136 participants, 76.5% (104/136) were male and the mean age was 40.5 (SD 9.9) years. The mean age of group B was significantly lower than group C (*P*=.01) (Table 1). There was a significant difference in the negative affect subscale of the MNWS between groups B and C (*P*=.02) and for insomnia between groups A and C (*P*=.01) (Table 2). Differences in other sociodemographic characteristics and smoking profile in the 3 RCT groups were not significant. More than 90% (128/136, 94.1%) had been prescribed free NRT in their smoking cessation treatment, but only 5 of 136 (3.7%) were prescribed varenicline (Table 2). There was also no significant difference in other smoking-related variables. Overall, 86.0% (117/136) of the participants had been abstinent for at least 28 days before joining the RCT with 81% (34/42) in group A, 95% (38/40) in group B, and 83% (45/54) in group C.

### Lapse and Relapse Rates

In the ITT analysis, fewer participants in group A reported smoking relapse than in group C at 2-month (17%, 7/42 vs 43%, 23/54; OR 0.27, 95% CI 0.10-0.71 *P*=0.008; power=74.6%) and 6-month (41%, 17/42 vs 61%, 33/54; OR 0.43, 95% CI 0.19-0.99, *P*=.049; power=54.9%) follow-ups. Also, the odds ratios of 2-month relapse rate adjusting for baseline difference in smoking urge and days of abstinence were significant (adjusted OR 0.26, 95% CI 0.09-0.74, *P*=.01) (Table 3). There was no significant difference in the relapse rate between groups B and C at 2-month (30%, 12/40 vs 43%, 23/54; OR 0.58, 95% CI 0.24-1.37, *P*=.21; power=36.9%) and 6-month (53%, 21/40 vs 61%, 33/54; OR 0.70, 95% CI 0.31-1.61, *P*=.40; power=20.6%) follow-ups. The power analysis showed that the comparison of the relapse rate between groups B and C had a large type II error (ie, accepting the null hypothesis when it was false). The odds ratios comparing 2-month self-reported relapse rate between groups A and C using ITT, LOCF (assumed to have the same smoking status as the last follow-up), and complete-case analysis were mostly significant and consistent.

**Table 1:** Sociodemographic and smoking characteristics of participants at entry to the smoking cessation clinics (N=136).

Baseline characteristics		Group A: WhatsApp n=42	Group B: Facebook n=40	Group C: control n=54	*P* ^a^	
					A vs C	B vs C
**Gender, n (%)**						
	Male	32 (76)	29 (73)	43 (80)	.69	.42
	Female	10 (24)	11 (28)	11 (20)		
Age (years), mean (SD)		40.4 (10.4)	37.6 (8.0)	42.7 (10.4)	.30	.01
**Marital status, n (%)**						
	Single	11 (26)	15 (38)	12 (22)	.73	.06
	Married	24 (57)	23 (58)	31 (57)		
	Other	6 (14)	2 (5)	11 (20)		
	Missing	1 (2)	0 (0)	0 (0)		
**Monthly personal income (HK$), n (%)**						
	<$10,000	10 (24)	8 (20)	14 (26)	.07	.13
	$10,000-$19,999	17 (41)	16 (40)	28 (52)		
	$20,000-$29,999	12 (29)	6 (15)	5 (9)		
	≥$30,000	2 (5)	8 (20)	6 (11)		
	Missing	1 (2)	2 (5)	1 (2.0)		
**FTND,** ^b^ **n (%)**						
	Mild	14 (33)	12 (30)	14 (26)	.61	.78
	Moderate	14 (33)	18 (45)	23 (43)		
	Severe	14 (33)	10 (25)	17 (32)		
Any quit attempt before intake, n (%)		35 (83)	31 (78)	36 (67)	.07	.25
Daily cigarette consumption, mean (SD)		14.5 (6.3)	15.3 (6.5)	17.1 (7.3)		

^a^ Chi-square test for categorical variables; *t* test for continuous variables.

^b^ FTND: Fagerstrom Test for Nicotine Dependence (1-3=mild, 4-5=moderate, 6-10=severe).

**Table 2:** Treatment condition and quitting characteristics of participants at baseline (N=136).

Treatment condition and quitting characteristics at baseline		Group A: WhatsApp n=42	Group B: Facebook n=40	Group C: control n=54	*P* ^a^	
					A vs C	B vs C
Had been prescribed NRT, n (%)		38 (91)	39 (98)	51 (94)	.43	.56
Had been prescribed varenicline, n (%)		2 (5)	0 (0)	3 (6)	.71	.22
**Frequency of smoking urge in past week, n (%)**					.11	.09
	Never	5 (12)	12 (30)	8 (15)		
	Occasionally	20 (48)	13 (33)	13 (24)		
	1-2 times per day	14 (33)	9 (23)	25 (46)		
	≥3 times per day	3 (7)	6 (15)	8 (15)		
**Intensity of smoking urge in past 24 hours, n (%)**					.40	.10
	No urge	14 (33)	19 (48)	16 (30)		
	Slight	18 (43)	17 (43)	27 (50)		
	Mild	8 (19)	3 (8)	11 (20)		
	Moderate/Severe	2 (5)	1 (3)	0 (0)		
**Frequency of thinking of the feeling of enjoying smoking, n (%)**					.62	.10
	Never	7 (17)	9 (23)	7 (13)		
	Seldom	18 (43)	20 (50)	19 (35)		
	Sometimes	15 (36)	9 (23)	25 (46)		
	Often	1 (2)	2 (5)	3 (6)		
	Very often	1 (2)	0 (0)	0 (0)		
**Minnesota Nicotine Withdrawal Scale-Chinese,** ^b^ **mean (SD)**						
	Negative affect	0.46 (0.49)	0.25 (0.53)	0.48 (0.69)	.44	.02
	Insomnia	0.39 (0.73)	0.60 (0.88)	0.64 (0.72)	.01	.43
**12-item Smoking Self-Efficacy (SEQ-12),** ^c^ **mean (SD)**						
	Internal stimuli	3.78 (0.94)	3.92 (1.02)	3.89 (0.78)	.75	.56
	External stimuli	4.03 (0.89)	4.11 (0.91)	4.11 (0.72)	.89	.43
Days of abstinence at baseline, mean (SD)		46.8 (16.3)	50.4 (10.5)	46.3 (16.9)	.64	.44
**Days of abstinence at baseline (category), n (%)**					.49	.26
	≤7 days	0 (0)	0 (0)	3 (6)		
	8-14 days	3 (7)	0 (0)	2 (4)		
	15-28 days	5 (12)	2 (5)	4 (7)		
	≥28 days	34 (81)	38 (95)	45 (83)		

^a^ Chi-square test for categorical variables; Mann-Whitney *U* test for continuous variables.

^b^ Greater values indicate stronger self-rated withdrawal symptoms.

^c^ Greater values indicate higher self-efficacy.

**Table 3:** Relapsed, lapsed, smoked in the past 7 days, and validated abstinence at 2- and 6-month follow-ups.

Quitting outcomes			Group, n (%)			Unadjusted OR (95% CI)		Adjusted OR^a^ (95% CI)	
			Group A (n=42)	Group B (n=40)	Group C (n=54)	A vs C	B vs C	A vs C	B vs C
**2-month follow-up**									
	**Relapse** ^b^								
		ITT	7 (17)	12 (30)	23 (43)	0.27 (0.10, 0.71)^e^	0.58 (0.24, 1.37)	0.26 (0.09, 0.74)^f^	0.47 (0.18, 1.25)
		LOCF	3 (7)	10 (25)	13 (24)	0.27 (0.07, 1.03)	1.17 (0.45, 3.05)	0.22 (0.05, 0.95)^f^	1.06 (0.35, 3.20)
		Complete case	2/37 (5)	10/38 (26)	13/43 (30)	0.15 (0.03, 0.71)^f^	0.92 (0.35, 2.47)	0.17 (0.04, 0.77)^f^	0.76 (0.24, 2.39)
	Lapse^c^		16 (38)	15 (38)	27 (50)	0.62 (0.27, 1.40)	0.60 (0.26, 1.38)	0.65 (0.27, 1.57)	0.58 (0.23, 1.46)
	Smoked in the past 7 days		7 (17)	12 (30)	24 (44)	0.25 (0.09, 0.66)^e^	0.54 (0.23, 1.27)	0.26 (0.09, 0.73)^f^	0.44 (0.17, 1.17)
	Validated abstinence^d^		16 (38)	15 (38)	13 (24)	1.94 (0.80, 4.69)	1.89 (0.77, 4.63)	1.66 (0.64, 4.33)	1.64 (0.61, 4.39)
**6-month follow-up**									
	**Relapse** ^b^								
		ITT	17 (41)	21 (53)	33 (61)	0.43 (0.19, 0.99)^f^	0.70 (0.31, 1.61)	0.35 (0.14, 0.86)^f^	0.73 (0.29, 1.83)
		LOCF	11 (26)	13 (33)	20 (37)	0.60 (0.25, 1.46)	0.82 (0.35, 1.94)	0.54 (0.21, 1.40)	0.75 (0.29, 1.96)
		Complete case	9/34 (27)	9/28 (32)	17/38 (45)	0.49 (0.19, 1.31)	0.59 (0.21, 1.62)	0.43 (0.15, 1.26)	0.57 (0.18, 1.79)
	Lapse^c^		24 (57)	22 (55)	33 (61)	0.85 (0.37, 1.93)	0.78 (0.34, 1.78)	0.71 (0.29, 1.74)	0.81 (0.32, 2.03)
	Smoked in the past 7 days		15 (36)	21 (53)	33 (61)	0.35 (0.15, 0.82)^f^	0.70 (0.31, 1.61)	0.29 (0.11, 0.72)^e^	0.68 (0.27, 1.71)
	Validated abstinence^d^		11 (26)	10 (25)	8 (15)	2.04 (0.74, 5.65)	1.92 (0.68, 5.41)	1.87 (0.62, 5.63)	2.01 (0.64, 6.36)

^a^ Odds ratio adjusted for age, frequency of smoking urge in past month, intensity of smoking urge in past 24 hours, and days of abstinence at baseline.

^b^ Relapse was defined as smoking 5 or more cigarettes in 3 consecutive days in the past 2 and 4 months at 2- and 6-month follow-ups, respectively. Intention-to-treat (ITT) analysis assumed participants who were lost to follow-up as relapsers or smokers. Last observation carried forward (LOCF) assumed participants who were lost to follow-up as the status of previous follow-up. Complete-case analysis excluded participants who were lost to follow-up.

^c^ Lapse was defined as any incidence of smoking in the past 2 and 4 months at 2- and 6-month follow-ups, respectively.

^d^ Validated abstinence (by ITT) was defined as self-reported abstinence validated by tests of exhaled carbon monoxide (≤4 ppm) and salivary cotinine (≤10 ng/mL).

^e^ *P*<.01.

^f^ *P*<.05.

Excluding the participants in groups A and B who did not participate in the social group (n=11), the corresponding significant odds ratios comparing groups A and C at 2- and 6-month follow-ups by ITT analysis confirmed the lower odds of relapse in group A (Multimedia Appendix 3). All odds ratios comparing the relapse rate between groups B and C were inconsistent and insignificant.

There was no significant difference in the lapse rate in all group comparisons at both follow-ups. Group A had a lower prevalence of 7-day self-reported smoking at 2-month (17%, 7/42 vs 44%, 24/54; OR 0.25, 95% CI 0.09-0.66, *P*=.005; power=83.7%) and 6-month (36%, 15/36 vs 61%, 33/61; OR 0.35, 95% CI 0.15-0.82, *P*=.02; power=67.6%) follow-ups. No significant difference between groups B and C was found. The 2-month participation rates of biochemical validation for the groups A, B, and C were 77% (20/26), 64% (16/25), and 32% (13/41), respectively. The corresponding figure at 6-month follow-up was 41% (11/27), 53% (10/19), and 33% (10/30). The prevalence of biochemically validated abstinence at both follow-ups in groups A and B were slightly higher than group C, but the difference was insignificant.

### Post Characteristics in the Social Groups

**Figure 2:**
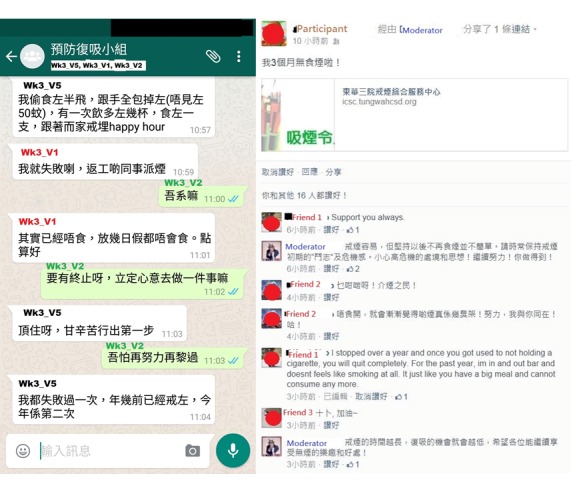
Screenshots of the WhatsApp (Left side) and Facebook (Right side) online social groups.

A total of 7 WhatsApp and 6 Facebook social groups were formed for groups A and B, respectively (Figure 2). Those who could not be added to the social groups (n=3) or left the social groups early (n=8) were considered “dropouts.” The number of dropouts in groups A and B was 7 of 42 (17%) and 4 of 40 (10%), respectively. The mean number of posts in the WhatsApp and Facebook social groups was 55.0 (SD 50.7) and 21.0 (SD 34.4), respectively. The WhatsApp social groups had more moderators’ posts (median 60, IQR 25 vs median 31.5, IQR 7; *P*=.05) and participants’ posts than Facebook (median 35, IQR 50 vs median 6, IQR 9; *P*=.07), but they did not meet statistical significance. In all, 23 of 42 (54.8%) WhatsApp participants posted 1 to 9 times in the social group (median 3, IQR 7), whereas approximately half (58%, 23/40) of the Facebook participants did not post any (median 0, IQR 2.3). One WhatsApp social group had only 5 participants’ posts because it had only 3 participants and 2 of them dropped out early. One Facebook social group had no posts from participants even though the moderator had already posted 24 posts of smoking cessation reminders. The majority of posts were sharing of smoking or quitting experiences (WhatsApp: 151/384 posts, 39.3%; Facebook: 81/123 posts, 65.9%) and simple reply to the moderator’s inquiry (WhatsApp: 131/384 posts, 34.1%; Facebook: 82/123 posts, 66.7%) (Table 4).

**Table 4:** Content analysis of the WhatsApp and Facebook social groups.

Posts characteristics		Group A: WhatsApp n=42	Group B: Facebook n=40
Number of social groups, n		7	6
Participants per social group, range		2-9	4-10
Participants who did not post anything, n (%)		5 (12)	11 (28)
Total moderators’ posts, n		465	255
Moderators’ posts per group, median (range)		60 (46-95)	31.5 (18-118)
Total participants’ posts, n		384	123
Participants’ posts per group, median (range)		35 (5-145)	6 (0-90)
**Participants’ posts per participant, n (%)**			
	0 or dropped out	10 (24)	23 (58)
	1-9	23 (55)	14 (35)
	10-19	7 (17)	2 (5)
	≥20	2 (5)	1 (3)
**Characteristic of the participants’ posts,** ^a^ **n (%)**			
	Sharing smoking/quitting experience	151 (39.3)	81 (65.9)
	Simple reply to moderator’s inquiry	131 (34.1)	82 (66.7)
	Self-reported lapse/relapse/maintaining abstinence	62 (16.1)	15 (12.2)
	Encouragement	44 (11.5)	12 (9.8)
	Reminders of quitting importance	24 (6.3)	4 (3.3)
	Suggesting methods for smoking cessation	21 (5.5)	7 (5.7)
	Seeking information related to smoking cessation and health	8 (2.1)	4 (3.3)
	Sharing information including pictures and videos	8 (2.1)	4 (3.3)
	Seeking help related to smoking cessation and health	4 (1.0)	2 (1.6)
	Others	85 (22.1)	4 (3.3)

^a^ For group A, total number of posts was 384; for group B, total number of posts was 123.

### Smoking-Related Variables at Follow-Ups

Daily smoking urge (*P=*.01), urge intensity (*P*=.02), mean score of enjoying smoking (*P*=.002), and the 2 subscales of the MNWS (*P*<.001) showed a decline significantly from baseline to 2-month follow-up and then remained steady at 6-month follow-up in the 3 RCT groups. The change of these variables in the 3 RCT groups was not significantly different (Multimedia Appendices 4-8). The mean score of the internal and external stimuli subscale of SEQ-12 increased at 2-month follow-up (*P*<.001), but fell at 6-month follow-up (Multimedia Appendices 9 and 10). Group A showed a greater increase in the internal stimuli score than group C (*P*=.04). Multimedia Appendix 11 shows the CONSORT-EHEALTH form.

## Discussion

### Summary of Findings

This pilot RCT found fewer participants in group A (WhatsApp) reported relapse than the control group at 2- and 6-month follow-ups. It was consistent with higher self-reported abstinence, greater change in the internal stimuli subscale of SEQ-12, and more moderators’ and participants’ posts in the social groups of group A. Group B (Facebook) and the control group had a similar relapse rate and the Facebook social groups had less posts than their WhatsApp counterparts.

### Interpretation

Our findings have shown that the group discussion and reminders via WhatsApp social groups for recent quitters significantly reduced relapse by 73% and 57% at 2 months and 6 months, respectively. The group discussion and reminders in the WhatsApp social groups could achieve a larger effect size of maintaining short-term abstinence than face-to-face group counseling [10,11,32]. This online platform with more interactions than the Facebook social groups increased social support and reduced relapse. Also, the reminders sent from the moderators were specially designed for relapse prevention of recent quitters. These results supported previous studies that an interactive text messaging service for preventing smoking relapse was effective and well accepted by recent quitters [14,15]. The WhatsApp social groups also enhanced tailored and immediate advice from counselors, which was beneficial for smoking cessation [21]. Further investigation of the conversation content and its association with abstinence is warranted. However, the intervention effect dissipated after the social groups closed. It suggests a longer intervention period might extend the effectiveness.

The Facebook group achieved an insignificantly lower relapse rate than the control group, which might be attributed to the insufficient sample size and statistical power. The lower effectiveness of the Facebook social groups might also be explained by the fewer moderator and participant posts than WhatsApp. Although Facebook social groups had the same standardized moderators’ reminders as WhatsApp, there was less interaction between the participants and between the participants and the moderator in the Facebook social groups than WhatsApp. Therefore, Facebook participants might receive less support in preventing smoking relapses. Such a difference might be due to the difference in usage habits between WhatsApp and Facebook users. Previous studies showed that Facebook supported dissemination of smoking cessation throughout the social network [33] and engaged a large number of smokers outside the social group [21]. However, some Facebook users used the platform with computers. They might post and reply less frequently than their WhatsApp counterparts, who could do so more frequently with a mobile phone. In addition, Facebook users might be distracted by other newsfeeds, which do not appear in WhatsApp. Our findings suggested that group discussion using online social media should consider some strategies to increase interactions, such as increasing interesting and attractive content and allowing other Facebook users to post in the social group.

The significant increase in self-efficacy in dealing with internal smoking cues such as bad mood and anxiety was found in the WhatsApp group only, which suggests that the interaction and peer support in the social groups were beneficial to manage these smoking cues. The association between the change in self-efficacy and relapse prevention warrants further exploration. However, the 3 RCT groups showed similar reduction in frequency and intensity of smoking urge, and withdrawal symptoms over the study period. This result might be due to the use of the NRT as smoking cessation treatment for the majority of participants. Also, because most had been abstinent for more than a month at baseline, their barriers of maintaining abstinence might not be smoking urge or withdrawal symptoms [34]. Future studies should test the effectiveness in unassisted quitters and quitters who had quit for a few days.

Approximately half of the participants reported smoking lapses at 6 months, which was similar to a recent exploratory study providing relapse prevention to recent quitters through text messages [14]. Our online social groups did not significantly reduce smoking lapses. It was consistent with their small change in dealing with those environmental smoking cues, as measured by the external stimuli subscale of the SEQ-12, and no interaction between group allocation and time. In turn, our intervention increased internal self-efficacy and enhanced instant feedback to the reported smoking lapses, which might effectively prevent the onset of a relapse following a lapse [35]. To improve the intervention for preventing smoking lapses, process evaluation of how it helped reduce smoking lapse is needed.

### Limitations

Several limitations should be noted. Firstly, the findings may only be generalizable to recent quitters (mostly male and married) who had received prior treatment from smoking cessation clinics. Such intervention should be further tested in different groups of smokers including unassisted quitters, alcoholics, and pregnant women. Secondly, most participants had already quit for a few weeks before joining the RCT, so the present RCT did not examine if the intervention helped participants manage smoking urges and withdrawal symptoms at the early stage of quitting. Thirdly, the pilot RCT had a small sample size so that the estimates of the odds ratios were not precise. Future studies with a larger sample size are warranted to confirm and quantify the effectiveness. Fourthly, differences in the smoking quantity at intake and urge frequency at baseline were found in the 3 RCT groups, which might be due to chance or no strict concealment of group allocation. Complete allocation concealment in the enrollment staff, if feasible, should be used in future RCTs. Lastly, the biochemically validated quit rate might be biased. Some self-reported quitters were too busy or perceived the validation unnecessary for them. Also, the incentive for participating in the validation might have motivated some self-reported quitters to maintain abstinence for the reward. Future studies should use other simple validation methods or compensation schemes to increase the participation in and validity of the validation.

### Conclusions

This pilot RCT has developed and provided the first preliminary evidence that group discussion and reminders via WhatsApp social group were effective to reduce smoking relapse. Clinical practice in using social networking services for relapse prevention should extend the intervention period, improve the intervention content to prevent smoking lapse and relapse, and increase interaction among participants. Future RCTs with larger sample sizes and unassisted smokers are warranted.

## Appendix

Multimedia Appendix 1 Using social networking service to prevent smoking relapse: Intervention guide.

Multimedia Appendix 2 Using social networking service to prevent smoking relapse: Moderator’s guideline.

Multimedia Appendix 3 Relapsed, lapsed, smoked in the past 7 days and validated abstinence at 2- and 6-month follow-up. (excluding 11 subjects in Group A and B who did not participate in the social groups).

Multimedia Appendix 4 Percentage of subjects who had daily smoking urge in the past week.

Multimedia Appendix 5 Mean score of intensity of smoking urge in the past 24 hours in quitters.

Multimedia Appendix 6 Frequency of thinking of enjoying smoking in the past month.

Multimedia Appendix 7 Minnesota Nicotine Withdrawal Scale (Chinese): Negative affect subscale.

Multimedia Appendix 8 Minnesota Nicotine Withdrawal Scale (Chinese): Insomnia subscale.

Multimedia Appendix 9 Smoking Self-efficacy: Internal Stimuli.

Multimedia Appendix 10 Smoking Self-efficacy: External Stimuli.

Multimedia Appendix 11 CONSORT-EHEALTH V1.6 form.

## Abbreviations

AUDIT: Alcohol Use Disorders Identification Test
ICSC: Integrated Centre of Smoking Cessation
ITT: intention-to-treat
LOCF: last observation carried forward
NRT: nicotine replacement therapy
RCT: randomized controlled trial
