# Supplementary material for: Using WhatsApp and Facebook Online Social Groups for Smoking Relapse Prevention for Recent Quitters: A Pilot Pragmatic Cluster Randomized Controlled Trial
Source: J Med Internet Res. 2015 Oct 22;17(10):e238. doi: 10.2196/jmir.4829 (PMC4642789; doi:10.2196/jmir.4829)
Supplement: Multimedia Appendix 1 [file jmir_v17i10e238_app1.pdf]

Multimedia Appendix 1

Using social networking service to prevent smoking relapse:

Intervention guide

Schedule and themes of moderator's posts:

	Mon	Tue	Wed	Thu	Fri	Sat	Sun
Week 1	1, 2		4		FU of 4		
Week 2	1, 2		4		FU of 4		
Week 3	1, 2		3		FU of 3		
Week 4	1, 2		3		FU of 3		
Week 5	1, 2		3		FU of 3		
Week 6	1, 2		5		FU of 5		
Week 7	1, 2		5		FU of 5		
Week 8	1, 2		6		FU of 6		

1 = Encourage to maintain abstinence; 2 = Importance of remaining abstinence; 3 = Prevent smoking triggers

4 = Handling withdrawal symptoms & lapse; 5 = Stress and mood management; 6 = Weight control

FU: Follow-up

Weekly contents:

Week 1

Monday - Greetings / Reminders and rules of the social group / Congratulation on quitting smoking

Wednesday – Information about withdrawal symptoms / how to withstand the symptoms / Video clip about Tips of quitting (http://www.e-quit.org/CustomPage/HtmlEditorPage.aspx?MId=699)

Friday - Ask the group members if they have encountered withdrawal symptoms

Week 2

Monday - Importance of remaining abstinence / Ask group members if they are abstinent / Encourage them to maintain abstinence / Mobile apps about smoking cessation created by Tobacco Control Office

Wednesday – Information about smoking lapse / Suggest coping strategies if lapse happens

Friday - Ask group members if they have experienced smoking lapse

Week 3

Monday - Importance of remaining abstinence / Ask group members if they are abstinent / Encourage to maintain abstinent / Video clip about quitting experience shared by an elderly ex-smoker (http://www.youtube.com/watch?v=XrVj9r65YrU)

Wednesday – Reminders about smoking cues that trigger smoking / Suggest tips of handling smoking cues

Friday – Ask group members if they have encountered smoking cues and how they have responded

Week 4

Monday - Importance of remaining abstinence / Ask group members if they are abstinent / Encourage to maintain abstinent / Website about an online smoking cessation aids (http://www.tco.gov.hk/iocc/index.html)

Wednesday - Reminders about smoking cues that trigger smoking / Suggest tips of handling smoking cues

Friday - Ask group members if they have encountered smoking cues and how they have responded

Week 5

Monday - Importance of remaining abstinence / Ask group members if they are abstinent / Encourage them to maintain abstinent / Video clip about two cases of successful quitting (http://www.youtube.com/watch?v=klFPoIdWgY0)

Wednesday – Reminders about habits that trigger smoking / how to kick out these habits

Friday - Ask group members if they have these habits and how they managed them

Week 6

Monday - Importance of remaining abstinence / Ask group members if they are abstinent / Encourage them to maintain abstinent

Wednesday – Remind them smoking is not good for coping emotional stress / Suggest methods of relieving stress / Introduce mobile apps of game for coping stress

Friday - Ask group members if they have stress and how they have coped stress

Week 7

Monday - Importance of remaining abstinence / Ask group members if they are abstinent / Encourage them to maintain abstinent

Wednesday – Explain why quitters have negative affect during abstinence / Suggest methods of coping negative affect / Video clip of a guided aerobic exercise (http://www.youtube.com/watch?v=Sm_hxY7KBvA)

Friday - Ask group members if they have negative emotion after quitting

Week 8

Monday - Importance of remaining abstinence / Ask group members if they are abstinent / Encourage them to maintain abstinent / A music video clip which have revised lyrics about quitting (http://www.youtube.com/watch?v=AghxMklLKVw)

Wednesday- Explain why quitters gain weight / Suggest methods of weight control / A website of diet for smoking cessation (http://www.e-quit.org/CustomPage/HtmlEditorPage.aspx?MId=572)

Friday - Ask group members if they gain weight
