# Supplementary material for: Using WhatsApp and Facebook Online Social Groups for Smoking Relapse Prevention for Recent Quitters: A Pilot Pragmatic Cluster Randomized Controlled Trial
Source: J Med Internet Res. 2015 Oct 22;17(10):e238. doi: 10.2196/jmir.4829 (PMC4642789; doi:10.2196/jmir.4829)
Supplement: Multimedia Appendix 2 [file jmir_v17i10e238_app2.pdf]

Multimedia Appendix 2

Using social networking service to prevent smoking relapse

Moderator's guideline

1. Routine tasks moderators need to do

- Create the discussion group and post contents according to the intervention guide.
- Reply the messages from the participants in two time slots (1-3pm and 6-8pm) on Monday to Saturday. Posting in other time is optional.
- Fill in the record sheet to keep a record of the discussion topics you have delivered every week.
- Copy and send the conversation contents of the discussion groups to the project coordinator (Vivian, viviwai@hku.hk) every two weeks.
- Remove all participants in the social group after the 2-month intervention. (Refer to guideline of using WhatsApp and Facebook P.11-13)

2. How to respond to the group members when discussing about smoking/quitting

- Initiate new topics of interest following the schedule of intervention guide. If a group member does not respond to any conversation for two weeks, ask him/her the reason. (*Remark the participant who did not have any response for at least two weeks)
- Initiate the conversation on Monday starting from week 1 to week 8 by asking whether participants have smoked in the past week. If a group member say "yes", moderator should follow up by asking "How many day(s) have you smoked in the past week?" and "How many cigarette(s) have you smoked on average in the smoking day?".
- Respond to the enquires about smoking cessation of the participants.
- Correct the myths and misunderstanding about smoking cessation of the participants with reference to the "Standardized reply to participants' enquiry".
- Summarize some key points of the discussion when necessary.

- If the participants send individual messages to the moderator to discuss the smoking/quitting issues, respond to their enquiries and encourage he/she to discuss in the group.
- Recommend the subjects to join the smoking cessation service of TWGHs again if they cannot handle the relapse situation.

3. How to deal with the group members with inappropriate actions in the groups

- Remove the posts which violate the regulation and explain in the group why it is deleted.
- Ignore the post that is outside the domain of this social networking groups and remind the participants by reminding them to post content related to smoking cessation

4. Other Rules

- Be the role model for the group and should not post offensive statement.
- Respect all group members and act in a manner that is considered polite, friendly, helpful and unbiased.
- Learn and be familiar with all of the regulations.
- Keep all the posts in the group as confidential and should not share and discuss the information in the group to others except the principle investigator, co-investigators and co-coordinators of the project.
- Group members have the right to withdraw from the social group. If any group member withdraws, moderator have to enquire the reasons and report the withdraw issue to the research coordinator.

Reference: http://festoolownersgroup.com/forum-rules-guidelines-suggestions/forum-moderator-guidelines/
