# Supplementary material for: Using WhatsApp and Facebook Online Social Groups for Smoking Relapse Prevention for Recent Quitters: A Pilot Pragmatic Cluster Randomized Controlled Trial
Source: J Med Internet Res. 2015 Oct 22;17(10):e238. doi: 10.2196/jmir.4829 (PMC4642789; doi:10.2196/jmir.4829)
Supplement: Multimedia Appendix 3 [file jmir_v17i10e238_app3.pdf]

Multimedia Appendix 3 Relapsed, lapsed, smoked in the past 7 days and validated abstinence at 2- and 6-month follow-up. (excluding 11 subjects in Group A and B who did not participate in the social groups)

	**Group A (N= 35) n (%)**	**Group B (N= 36) n (%)**	**Group C (N= 54) n (%)**	**Unadjusted OR A versus C (95%CI)**	**Unadjusted OR B versus C (95%CI)**	**Adjusted OR[a] A versus C (95%CI)**	**Adjusted OR[a] B versus C (95%CI)**
**2-month follow-up**							
Relapse[b]							
ITT[c]	6 (17.1)	11 (30.6)	23 (42.6)	0.28 (0.10, 0.78)*	0.59 (0.24, 1.45)	0.27 (0.09, 0.83)*	0.45 (0.16, 1.24)
LOCF[d]	3 (8.6)	10 (27.8)	12 (22.2)	0.33 (0.09, 1.26)	1.35 (0.51, 3.56)	0.26 (0.06, 1.15)	1.18 (0.39, 3.60)
CC[e]	3/32 (9.4)	10/35 (28.6)	12/43 (27.9)	0.27 (0.07, 1.04)	1.03 (0.38, 2.78)	0.21 (0.05, 0.96)*	0.82 (0.26, 2.59)
Lapse[f]	14 (40.0)	13 (36.1)	27 (50.0)	0.67 (0.28, 1.58)	0.57 (0.24, 1.34)	0.75 (0.30, 1.92)	0.52 (0.20, 1.37)
Smoked in the past 7 days	6 (17.1)	11 (30.6)	24 (44.4)	0.26 (0.09, 0.72)*	0.55 (0.23, 1.34)	0.27 (0.09, 0.83)*	0.42 (0.15, 1.16)
Validated abstinence[g]	15 (42.9)	14 (38.9)	13 (24.1)	2.37 (0.95, 5.91)	2.01 (0.80, 5.01)	2.08 (0.76, 5.69)	1.95 (0.71, 5.39)
**6-month follow-up**							
Relapse[b]							
ITT[c]	13 (37.1)	18 (50.0)	33 (61.1)	0.38 (0.16, 0.90)*	0.64 (0.27, 1.49)	0.28 (0.10, 0.76)*	0.68 (0.26, 1.76)
LOCF[d]	9 (25.7)	12 (33.3)	20 (37.0)	0.59 (0.23, 1.50)	0.85 (0.35, 2.06)	0.51 (0.18, 1.42)	0.82 (0.31, 2.20)
CC[e]	8/30 (26.7)	8/26 (30.8)	17/38 (44.7)	0.45 (0.16, 1.26)	0.55 (0.19, 1.57)	0.36 (0.11, 1.16)	0.57 (0.17, 1.91)
Lapse[f]	20 (57.1)	19 (52.8)	33 (61.1)	0.85 (0.36, 2.01)	0.71 (0.30, 1.67)	0.69 (0.26, 1.79)	0.76 (0.29, 1.97)
Smoked in the past 7 days	12 (34.3)	18 (50)	33 (61.1)	0.33 (0.14, 0.81)*	0.64 (0.27, 1.49)	0.26 (0.10, 0.70)*	0.65 (0.25, 1.67)
Validated abstinence[g]	10 (28.6)	10 (27.8)	8 (14.8)	2.30 (0.81, 6.57)	2.21 (0.78, 6.30)	2.09 (0.67, 6.56)	2.33 (0.73, 7.42)

*p<.05; **p<.01; Bold figures indicate values with moderate or strong effect size

[a] Odds ratio adjusted for age, frequency of smoking urge in past month, intensity of smoking urge in past 24 hours and days of abstinence at baseline.

[b] Relapse is defined as smoking 5 or more cigarettes in 3 consecutive days in the past 2 and 4 months at 2- and 6-month follow-up, respectively.

[c] Intention-to-treat (ITT) analysis assumed subjects who were lost to follow-up as relapsers

[d] Last-observation-carried-forward (LOCF) assumed subjects who were lost to follow-up as the status of previous follow-up

[e] Complete case (CC) analysis excluded subjects who were lost to follow-up

[f] Lapse is defined as any incidence of smoking in the past 2 and 4 months at 2- and 6-month follow-up, respectively.
[g] Validated abstinence is defined as self-reported abstinence validated by tests of exhaled carbon monoxide (≤4ppm) and salivary cotinine (≤10ng/ml).
