# Supplementary material for: Using WhatsApp and Facebook Online Social Groups for Smoking Relapse Prevention for Recent Quitters: A Pilot Pragmatic Cluster Randomized Controlled Trial
Source: J Med Internet Res. 2015 Oct 22;17(10):e238. doi: 10.2196/jmir.4829 (PMC4642789; doi:10.2196/jmir.4829)
Supplement: Multimedia Appendix 4 [file jmir_v17i10e238_app4.pdf]

Multimedia Appendix 4 Percentage of subjects who had daily smoking urge in the past week.

General linear model repeated measures analysis: Time effect P <.01; Group effect (A versus C) P = .04; Group effect (B versus C) P = .22; Interaction of time and group (A versus C) P = .70; Interaction of time and group (B versus C) P = .80
