# Supplementary material for: Using WhatsApp and Facebook Online Social Groups for Smoking Relapse Prevention for Recent Quitters: A Pilot Pragmatic Cluster Randomized Controlled Trial
Source: J Med Internet Res. 2015 Oct 22;17(10):e238. doi: 10.2196/jmir.4829 (PMC4642789; doi:10.2196/jmir.4829)
Supplement: Multimedia Appendix 5 [file jmir_v17i10e238_app5.pdf]

Multimedia Appendix 5 Mean score of intensity of smoking urge in the past 24 hours in quitters

Remark: 0=Not present, 1=Slight, 2=Mild, 3=Moderate, 4=Severe
General linear model repeated measures analysis: Time effect *P* = .02; Group effect (A versus C) *P* = .33; Group effect (B versus C) *P* = .01; Interaction of time and group (A versus C) *P* = .32; Interaction of time and group (B versus C) *P* =.57
