# Supplementary material for: Using WhatsApp and Facebook Online Social Groups for Smoking Relapse Prevention for Recent Quitters: A Pilot Pragmatic Cluster Randomized Controlled Trial
Source: J Med Internet Res. 2015 Oct 22;17(10):e238. doi: 10.2196/jmir.4829 (PMC4642789; doi:10.2196/jmir.4829)
Supplement: Multimedia Appendix 6 [file jmir_v17i10e238_app6.pdf]

Multimedia Appendix 6 Frequency of thinking of enjoying smoking in the past month.

Remark: 0 = Never, 1 = Occasionally, 2 = 1-2 times per day, 3 = 3-9 times per day, 4 = 10 times or more per day

General linear model repeated measures analysis: Time effect $P < .01$; Group effect (A versus C) $P = .03$; Group effect (B versus C) $P = .96$; Interaction of time and group (A versus C) $P = .32$; Interaction of time and group (B versus C) $P = .48$
