# Supplementary material for: Using WhatsApp and Facebook Online Social Groups for Smoking Relapse Prevention for Recent Quitters: A Pilot Pragmatic Cluster Randomized Controlled Trial
Source: J Med Internet Res. 2015 Oct 22;17(10):e238. doi: 10.2196/jmir.4829 (PMC4642789; doi:10.2196/jmir.4829)
Supplement: Multimedia Appendix 7 [file jmir_v17i10e238_app7.pdf]

Multimedia Appendix 7 Minnesota Nicotine Withdrawal Scale (Chinese): Negative affect subscale.

Remark: The Negative Affect subscale included the intensity of (1) bad mood, (2) easily get angry/frustrated, (3) feel anxious and (4) difficult to concentrate; 0= Never, 1= Mild, 2= Moderate, 3= Severe, 4= Very severe

General linear model repeated measures analysis: Time effect $P < .01$; Group effect (A versus C) $P = .44$; Group effect (B versus C) $P = .96$; Interaction of time and group (A versus C) $P = .75$; Interaction of time and group (B versus C) $P = .85$
