# Supplementary material for: Using WhatsApp and Facebook Online Social Groups for Smoking Relapse Prevention for Recent Quitters: A Pilot Pragmatic Cluster Randomized Controlled Trial
Source: J Med Internet Res. 2015 Oct 22;17(10):e238. doi: 10.2196/jmir.4829 (PMC4642789; doi:10.2196/jmir.4829)
Supplement: Multimedia Appendix 8 [file jmir_v17i10e238_app8.pdf]

Multimedia Appendix 8 Minnesota Nicotine Withdrawal Scale (Chinese): Insomnia subscale.

Remarks: The Insomnia subscale included the intensity of (1) difficult to sleep and (2) easily awaken at night; 0= Never, 1= Mild, 2= Moderate, 3= Severe, 4= Very severe
General linear model repeated measures analysis: Time effect $P < .01$; Group effect (A versus C) $P = .61$; Group effect (B versus C) $P = .25$; Interaction of time and group (A versus C) $P = .56$; Interaction of time and group (B versus C) $P = .20$
