# Supplementary material for: Using WhatsApp and Facebook Online Social Groups for Smoking Relapse Prevention for Recent Quitters: A Pilot Pragmatic Cluster Randomized Controlled Trial
Source: J Med Internet Res. 2015 Oct 22;17(10):e238. doi: 10.2196/jmir.4829 (PMC4642789; doi:10.2196/jmir.4829)
Supplement: Multimedia Appendix 9 [file jmir_v17i10e238_app9.pdf]

Multimedia Appendix 9 Smoking Self-efficacy: Internal Stimuli.

Remarks: The Internal Stimuli subscale included the self-efficacy in the following situations (1) Feeling anxious; (2) Getting angry; (3) Feeling nervous; (4) Bad mood; (5) Thinking about a difficult problem; 1= Absolutely not sure not to smoke, 2= Not sure not to smoke, 3= Approximately sure not to smoke, 4= Sure not to smoke, 5= Absolutely not to smoke

General linear model repeated measures analysis: Time effect $P < .01$; Group effect (A versus C) $P = .24$; Group effect (B versus C) $P = .56$; Interaction of time and group (A versus C) $P = .04$; Interaction of time and group (B versus C) $P = .83$
