# Supplementary material for: Using WhatsApp and Facebook Online Social Groups for Smoking Relapse Prevention for Recent Quitters: A Pilot Pragmatic Cluster Randomized Controlled Trial
Source: J Med Internet Res. 2015 Oct 22;17(10):e238. doi: 10.2196/jmir.4829 (PMC4642789; doi:10.2196/jmir.4829)
Supplement: Multimedia Appendix 10 [file jmir_v17i10e238_app10.pdf]

Multimedia Appendix 10 Smoking Self-efficacy: External Stimuli.

Remarks: The External Stimuli subscale included the self-efficacy in the following situations (1) Feeling the urge to smoke; (2) with smokers; (3) Having a drink with friend; (4) Celebrating something (5) Drinking beer, wine or other spirits; (6) After a meal; and (7) Having coffee or tea; 1= Absolutely not sure not to smoke, 2= Not sure not to smoke, 3= Approximately sure not to smoke, 4= Sure not to smoke, 5= Absolutely not to smoke

General linear model repeated measures analysis: Time effect $P < .01$; Group effect (A versus C) $P = .34$; Group effect (B versus C) $P = .75$; Interaction of time and group (A versus C) $P = .26$; Interaction of time and group (B versus C) $P = .36$
